# Dynamics of gut microbiota during pregnancy in women with TPOAb-positive subclinical hypothyroidism: a prospective cohort study

**DOI:** 10.1186/s12884-022-04923-5

**Published:** 2022-07-26

**Authors:** Min Wu, Cheng Chi, Yuxi Yang, Shan Guo, Tianhe Li, Muqing Gu, Tingting Zhang, Huimin Gao, Ruixia Liu, Chenghong Yin

**Affiliations:** 1 Department of Internal Medicine, Beijing Obstetrics and Gynecology Hospital, Capital Medical University, Beijing Maternal and Child Health Care Hospital, Beijing, 100026 China; 2 School of Nursing, Jining Medical University, Jining, 272067 China; 3 Department of Central Laboratory, Beijing Obstetrics and Gynecology Hospital, Capital Medical University, Beijing Maternal and Child Health Care Hospital, Beijing, 100026 China

**Keywords:** Subclinical hypothyroidism, Pregnancy, Anti-thyroid peroxidase antibody, Gut microbiota, Second trimester, Third trimester, Levothyroxine

## Abstract

**Background:**

Anti-thyroid peroxidase antibody (TPOAb) positivity can contribute to inhibit thyroxine synthesis. Gut microbiota can interact with metabolic or immune diseases. However, dynamics of gut microbiota from the second (T2) to the third trimester (T3) in women with TPOAb-positive/negative subclinical hypothyroidism (TPOAb^+^/TPOAb^−^ SCH) have not been reported. Therefore, we aimed to evaluate whether gut microbiota can be potential therapeutic targets for managing TPOAb^+^ SCH.

**Methods:**

In this single-center prospective cohort study, we observed gut microbiota dynamics by sequencing 16S rRNA from fecal samples collected in T2 (20–23^+ 6^ weeks) and T3 (28–33^+ 6^ weeks). TPOAb^+^/TPOAb^−^ SCH were stratified depending on whether or not they used levothyroxine (LT_4_) during the pregnancy (LT_4_^+^/LT_4_^−^). Microbiome bioinformatics analyses were performed using QIIME2. The linear discriminant analysis effect size (LEfSe) was used for the quantitative analysis of biomarkers. Functional profiling was performed with PICRUSt2.

**Results:**

Distinct gut microbiota dynamics from T2 to T3 were noted in the TPOAb^−^ (*n* = 68) and TPOAb^+^ (*n* = 64) SCH groups. The TPOAb^+^ LT_4_^−^ group was characterized by enriched bacterial amplicon sequence variants (ASVs) of *Prevotella* in T2 and *Bacteria*, *Lachnospirales*, *Lachnospiraceae*, *Blautia*, and *Agathobacter* in T3 and by depleted ASVs of *Gammaproteobacteria*, *Enterobacterales*, and *Enterobacteriaceae* in T2 and *Actinobacteriota*, *Coriobacteriia*, *Actinobacteria*, *Coriobacteriales*, *Bifidobacteriales*, *Bifidobacteriaceae*, *Bifidobacterium, Dorea formicigenerans*, and *Bifidobacterium longum* in T3. The TPOAb^+^ LT_4_^+^ group was characterized by enriched bacterial ASVs of *Blautia, Streptococcus salivarius*, and *Bifidobacterium longum* in T3 and by depleted ASVs of *Bacteroidota*, *Bacteroidia*, *Bacteroidales*, and *Prevotella* in T2 and *Agathobacter* in T3. Moreover, we identified 53 kinds of metabolic functions that were mainly involved in sugar, lipid, and amino acid metabolism.

**Conclusions:**

Our results indicated that low dynamics of gut microbiota composition and high dynamics of its metabolic function from T2 to T3 were associated with TPOAb^+^ SCH. We concluded that gut microbiota could be new targets for treatment of TPOAb^+^ SCH during pregnancy.

**Trial registration:**

This study was retrospectively registered at the Chinese Clinical Trial Registry (registration number ChiCTR2100047175) on June 10, 2021.

**Supplementary Information:**

The online version contains supplementary material available at 10.1186/s12884-022-04923-5.

## Background

Based on the 2017 American Thyroid Association (ATA) guidelines, subclinical hypothyroidism (SCH) in pregnancy refers to the elevation of thyroid stimulating hormone (TSH) level with normal free T_4_ (FT_4_) levels [1]; this occurs in 3–8% of women during the child-bearing period [2]. The 2017 ATA guidelines [1] recommend the establishment of trimester-specific reference ranges of serum TSH levels. The prevalence of SCH during pregnancy varies because of differences in trimester-specific reference ranges of TSH levels [3–5]. Numerous studies have associated SCH with increased adverse pregnancy and negative perinatal outcomes, including gestational hypertension, placental abruption, preterm delivery, fetal distress, neonatal death, and intrauterine growth restriction [2, 6–9]. One third of women with SCH have been reported to show positivity for anti-thyroid peroxidase antibody (TPOAb^+^) [10]. Studies in this discipline have also evidenced that TPOAb^+^ SCH is linked with adverse outcomes, including spontaneous abortion, preterm birth, and poor neural development of offsprings [11–14]. The 2017 ATA guidelines recommend that SCH women be prescribed levothyroxine (LT_4_) supplementation treatment for the prevention of abortion and preterm birth, regardless of TPOAb status [1]. Specifically, some studies have reported that LT_4_ treatment could reduce the chances of abortion and preterm delivery among pregnant women with TPOAb^+^ SCH [15, 16].

Intestinal microbiota, which include millions of microorganisms, sustain homeostasis by interacting with the host [17, 18]. Furthermore, metabolites of intestinal bacteria, such as short chain fatty acids (SCFAs), can impact intestinal barrier and signaling pathways [19]; this further affects the absorption of microelements, bile acid, deiodinase, and glucuronic acid (which jointly maintain normal thyroid function) [20].

In most studies, women with TPOAb^+^/TPOAb-negative (TPOAb^−^) SCH have been enrolled in the first trimester (T1). However, the dynamics of gut microbiota during the second (T2) and third trimesters (T3) in women with TPOAb^+^/TPOAb^−^ SCH have not been reported. In this single-center, prospective observational cohort study, we aim to observe the differences in the dynamics of gut microbiota composition and metabolic function from T2 to T3 in pregnant women with TPOAb^+^ and TPOAb^−^ SCH. To achieve this, we performed 16S rRNA sequencing of fecal samples of 64 and 68 women with TPOAb^+^ and TPOAb^−^ SCH, respectively. This study can show whether intestinal microbiota could be new targets for the treatment of TPOAb^+^ SCH during pregnancy.

## Methods

### Study population

This nested, prospective observational cohort study was conducted in the Beijing Obstetrics and Gynecology Hospital, Capital Medical University between June 2020 and May 2021. This study was approved by the Ethics Committee of the Beijing Obstetrics and Gynecology Hospital (No. 2018-KY-003-01, 2018-KY-003-02). All participants provided written informed consent. This study was recorded at the Chinese Clinical Trial Registry (registration number ChiCTR2100047175). All procedures conformed to the Declaration of Helsinki.

Inclusion criteria were as follows: (1) presence of singleton pregnancy; (2) recruitment at a gestational age of 6–13^+ 6^ weeks; (3) diagnosis of SCH based on thyroid function testing during T1; and (4) provision of informed consent.

Exclusion criteria were as follows: (1) occurrence of abortion or loss to follow-up; (2) history of other severe systemic autoimmune diseases; (3) history of severe heart, liver, kidney, lung, and/or other organ dysfunctions; (4) random adjustments to the daily dose of LT_4_; (5) failure to collect a fecal sample during T2 or T3; (6) use of antibiotics or probiotics 1 month prior to the collection of the fecal sample; (7) use of medications that affect thyroid function; (8) presence of endemic goiter; or (9) history of mental illness.

### Group design

In T1, pregnant women were screened for thyroid function, according to China’s Guidelines for the Diagnosis and Treatment of Thyroid Diseases, Pregnancy and Postpartum (Second Edition), 2019. Serum FT_4_ (enzyme immunoassay), TSH3UL (enzyme immunoassay), and TPOAb levels were detected using an automatic chemiluminescence immunoanalyzer (CENTAUR XP, Siemens, USA). The women’s clinical chemistry and hemoglobin levels were monitored with an automatic biochemical analyzer (CI16200, Abbott, USA) and a blood cell analyzer (XN2000, Sysmex, Japanese), respectively.

TPOAb^+^ SCH was defined by a TSH3UL level > 3.56 mIU/L, an FT_4_ level within the range of 11.80–18.40 pmol/L, and a TPOAb level > 60.00 U/mL. TPOAb^−^ SCH was defined by a TSH3UL level > 3.56 mIU/L, an FT_4_ level within the range of 11.80–18.40 pmol/L, and a TPOAb level within the range of 0.00–60.00 U/mL. TPOAb^+^/TPOAb^−^ women with SCH were stratified according to whether or not they were administered LT_4_ treatment during pregnancy (LT_4_^+^ or LT_4_^−^, respectively) (Supplementary Fig. 1).

### Fecal sample collection

Fecal samples were collected in T2 (20–23^+ 6^ weeks) and T3 (28–33^+ 6^ weeks) and analyzed using the PSP® Spin Stool DNA Plus Kit (SARSTEDT, Germany). Pregnant women collected their fecal samples in clean plastic bags after urination. Duplicate samples from the middle of the stool were preserved in individual sterile tubes. Fecal samples were transported to the hospital on the day they were collected and stored at − 80 °C until analysis.

### 16S rRNA amplicon sequencing and analysis

Total genomic DNA was extracted using the sodium dodecyl sulfate [SDS] and cetyltrimethyl ammonium bromide [CTAB] methods, according to the manufacturer’s instructions. The hypervariable V3–V4 regions of the bacterial 16S rRNA genes were amplified using the following primers: 341F (5′-CCTAYGGGRBGCASCAG-3′) and 806R (5′-GGACTACNNGGGTATCTAAT-3′). All PCR reactions were performed under the following conditions: 15 μL of Phusion® High-Fidelity PCR Master Mix (New England Biolabs), 0.2 μM of forward and reverse primers, and approximately 10 ng template DNA. Thermal cycling involved initial denaturation at 98 °C for 1 min, followed by 30 cycles of denaturation at 98 °C for 10 s, annealing at 50 °C for 30 s, extension at 72 °C for 30 s, and finally elongation at 72 °C for 5 min. PCR products were detected with 2% agarose gel electrophoresis. PCR products were mixed in equidensity ratios and purified using the Qiagen Gel Extraction Kit (Qiagen, Germany). Sequencing libraries were generated using the TruSeq® DNA PCR-Free Sample Preparation Kit (Illumina, USA), and index codes were added. Library quality was assessed using a Qubit@ 2.0 Fluorometer (Thermo Scientific) and an Agilent Bioanalyzer 2100 system. The library was sequenced on an Illumina NovaSeq platform and 250 bp paired-end reads were generated.

Microbiome bioinformatics analyses were carried out with QIIME2 (2021.04) [21]. Using the DADA2 plugin, sequences were quality filtered, denoised, and merged; chimeras were removed [22]. Species annotation was performed using QIIME2. On the basis of the Silva Database (Release138, https://www.arb-silva.de) [23], we performed 16S annotation. Non-parametric Kruskal–Wallis sum-rank test was used to analyze differences in α-diversity indices and similarity distance among different groups. The α-diversity indices (Chao1, Shannon, Simpson, and Abundance-based Coverage Estimator [ACE]) were calculated with QIIME2 and displayed with R software. The β-diversity was calculated using unweighted and weighted unifrac with QIIME2. The PerMANOVA analysis was performed on the distance matrices to illustrate the significance of β-diversity analysis. A matrix of unweighted or weighted unifrac distances was transformed into a new set of orthogonal axes, where the maximum variation factor was demonstrated by the first principal coordinate axis (PCoA1), and the second maximum variation factor was demonstrated by the second principal coordinate axis (PCoA2). The two-dimensional PCoA results were displayed using the ade package and ggplot2 package in R (Version 3.6.2). The linear discriminant analysis (LDA) effect size (LEfSe) (Online tool address http://huttenhower.sph.harvard.edu/galaxy/) [24] was used for the quantitative analysis of biomarkers (LDA score threshold: 2 or 4). Functional profiling was performed using Phylogenetic Investigation of Communities by Reconstruction of Unobserved States (PICRUSt2) (Online tool address https://github.com/picrust/picrust2) [25] with a single script (PICRUSt2_pipeline. Py).

### Statistical analysis of clinical data

EpiData was used for double data entry and validation. SPSS 26.0 software was used for statistical analysis of clinical data. Normally and non-normally distributed continuous variables are reported as means ± standard deviations and as medians with quartiles, respectively, and were compared using the independent samples Student’s t-test and Wilcoxon signed-rank test, respectively. Categorical variables are reported as frequency [n (%)]. Rank categorical variables were compared using the Wilcoxon signed-rank test; other categorical variables were compared using the chi-square test. *P* < 0.05 was considered to indicate a statistically significant difference.

## Results

### Clinical characteristics of subjects

A total of 64 and 68 women with TPOAb^+^ and TPOAb^−^ SCH, respectively, during pregnancy were included in this study. Women with TPOAb^+^ SCH were more likely to have a history of thyroid disease (37.5% vs. 14.7%, *P* = 0.003) and significantly higher T1 levels of total cholesterol (4.34 mmol/L vs. 3.98 mmol/L, *P* = 0.009), high-density lipoprotein-cholesterol (1.52 mmol/L vs. 1.39 mmol/L, *P* = 0.037), and low-density lipoprotein-cholesterol (2.38 mmol/L vs. 2.08 mmol/L, *P* = 0.036) than those with TPOAb^−^ SCH. Other clinical characteristics were not significantly different between groups (Table 1).

**Table 1:** Demographic and clinical characteristics of the study participants

Characteristic	TPOAb-positive women with SCH (64 cases)	TPOAb-negative women with SCH (68 cases)	*P* value
**General information**			
Han ethnicity, *n* (%)	59 (92.2)	62 (91.2)	0.834
Education background (postgraduate and above), *n* (%)	15 (23.4)	17 (25.0)	0.996
Education background (undergraduate), *n* (%)	35 (54.7)	35 (51.5)	
Education background (college and below), *n* (%)	14 (21.9)	16 (23.5)	
Family income (over 4 × 10^5^yuan/year), *n* (%)	20 (31.3)	17 (25.0)	0.736
Family income (10^5^to 4 × 10^5^yuan/year), *n* (%)	36 (56.2)	45 (66.2)	
Family income (less than 10^5^ yuan/year), *n* (%)	8 (12.5)	6 (8.8)	
First pregnancy, *n* (%)	36 (56.3)	38 (55.9)	0.966
Thyroid disease history, *n* (%)	24 (37.5)	10 (14.7)	**0.003**
Natural pregnancy, *n* (%)	62 (96.9)	64 (94.1)	0.681
Smoking, *n* (%)	4 (6.3)	4 (5.9)	1.000
Drinking, *n* (%)	4 (6.3)	2 (2.9)	0.430
**Indicator in the first trimester**			
Sickness, *n* (%)	21 (32.8)	32 (47.1)	0.095
Animals exposure, *n* (%)	8 (12.5)	13 (19.1)	0.299
Age (year), median (IQR)	33 (30–36)	33 (31–34)	0.913
BMI (kg/m^2^), median (IQR)	21.8 (20.2–24.6)	22.0 (19.9–25.2)	0.956
SBP (mmHg), mean ± SD	112 ± 11	110 ± 10	0.171
DBP (mmHg), median (IQR)	66 (58–74)	65 (57–70)	0.440
ALT(U/L), median (IQR)	12.35 (9.73–21.50)	12.25 (10.03–18.15)	0.816
AST(U/L), median (IQR)	14.45 (13.05–17.15)	15.40 (13.35–16.98)	0.347
ALB(g/L), median (IQR)	43.80 (42.33–45.78)	43.85 (42.30–45.00)	0.437
GLU (mmol/L), median (IQR)	4.64 (4.51–4.92)	4.67 (4.44–4.85)	0.537
BUN (mmol/L), median (IQR)	2.97 (2.67–3.53)	3.05 (2.56–3.39)	0.375
UA (μmol/L), median (IQR)	216.65 (187.25–257.63)	216.55 (187.53–254.28)	0.975
CRE (μmol/L), mean ± SD	49.43 ± 6.64	48.53 ± 5.79	0.403
TC (mmol/L), median (IQR)	4.34 (3.85–4.86)	3.98 (3.63–4.40)	**0.009**
TG (mmol/L), median (IQR)	0.94 (0.67–1.40)	1.04 (0.74–1.49)	0.631
HDL-C (mmol/L), median (IQR)	1.52 (1.33–1.75)	1.39 (1.23–1.57)	**0.037**
LDL-C (mmol/L), median (IQR)	2.38 (1.93–2.86)	2.08 (1.82–2.48)	**0.036**
HGB(g/L), median (IQR)	130 (120–138)	131 (123–136)	0.913

*Abbreviations*: *IQR* Interquartile range, *TPOAb* Thyroid peroxidase antibody, *BMI* Body mass index, *SBP* Systolic blood pressure, *DBP* Diastolic blood pressure, *ALT* Alanine aminotransferase, *AST* Aspartic acid aminotransferase, *ALB* Albumin, *GLU* Blood glucose, *BUN* Blood urea nitrogen, *UA* Uric acid, *CRE* Creatinine, *TC* Total cholesterol, *TG* Triglycerides, *HDL-C* High-density lipoprotein-cholesterol, *LDL-C* Low-density lipoprotein-cholesterol, *HGB* Hemoglobin

Stratification according to LT_4_ treatment status during pregnancy was as follows: TPOAb^+^ LT_4_^−^ (AZ1 or AW1) group, 8 women in T2 or T3; TPOAb^−^ LT_4_^−^ (BZ1 or BW1) group, 18 women in T2 or T3; TPOAb^+^ LT_4_^+^ (AZ2 or AW2) group, 56 women in T2 or T3; and TPOAb^−^ LT_4_^+^ (BZ2 or BW2) group, 50 women in T2 or T3 (Supplementary Fig. 1).

### Composition of gut microbiota

**Fig. 1:**
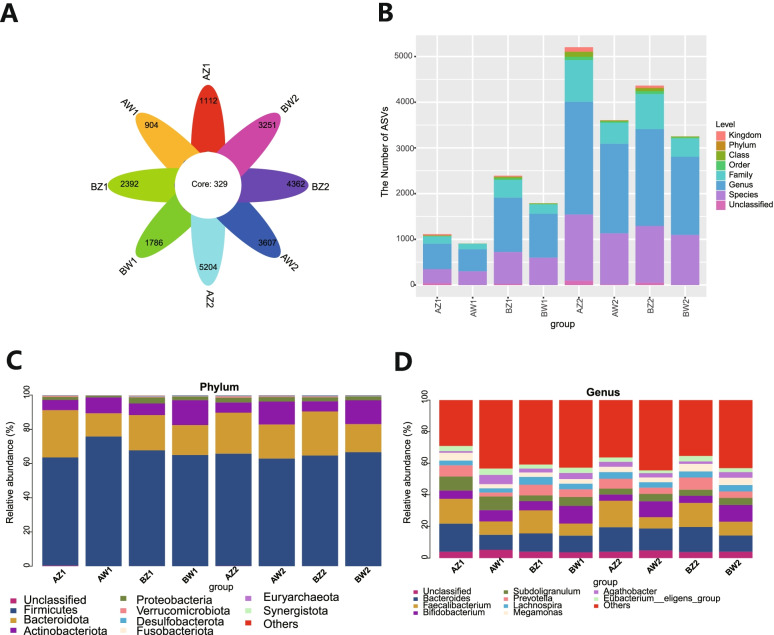
Flower diagram, bar chart and taxonomic analysis. **A** Flower diagram showing the number of common and unique amplicon sequence variants (ASVs) among the eight subgroups. Different subgroups are represented by different colors. Petals indicate the ASVs unique to each subgroup. The core shows the common ASVs across the subgroups. **B** Bar chart showing a number of ASVs classified to the family, genus, and species levels. **C**-**D** Taxonomic analysis. AZ1, TPOAb^+^-LT_4_^−^-T2, women in T2 with TPOAb-positive SCH and no LT_4_ treatment; AW1, TPOAb^+^-LT_4_^−^-T3, women in T3 with TPOAb-positive SCH and no LT_4_ treatment; BZ1, TPOAb^−^-LT_4_^−^-T2, women in T2 with TPOAb-negative SCH and no LT_4_ treatment; BW1, TPOAb^−^-LT_4_^−^-T3, women in T3 with TPOAb-negative SCH and no LT_4_ treatment; AZ2, TPOAb^+^-LT_4_^+^-T2, women in T2 with TPOAb-positive SCH and LT_4_ treatment; AW2, TPOAb^+^-LT_4_^+^-T3, women in T3 with TPOAb-positive SCH and LT_4_ treatment; BZ2, TPOAb^−^-LT_4_^+^-T2, women in T2 with TPOAb-negative SCH and LT_4_ treatment; BW2, TPOAb^−^-LT_4_^+^-T3, women in T3 with TPOAb-negative SCH and LT_4_ treatment

**Fig. 2:**
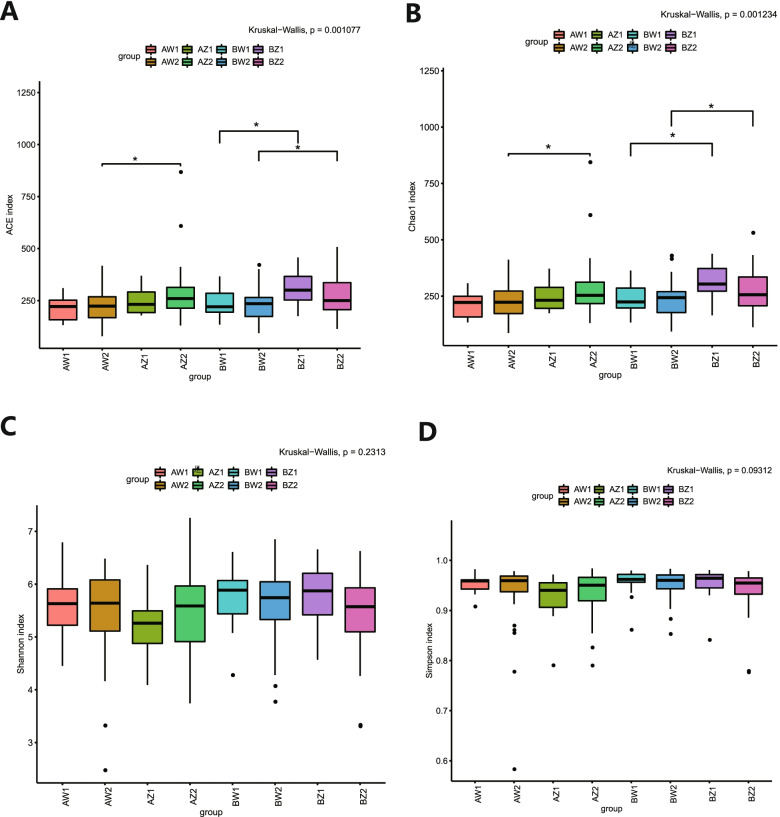
The α-diversity analysis. **A**-**D** α-diversity index (ACE, Chao1, Shannon, and Simpson) analysis. * *P* < 0.05. AZ1, TPOAb^+^-LT_4_^−^-T2; AW1, TPOAb^+^-LT_4_^−^-T3; BZ1, TPOAb^−^-LT_4_^−^-T2; BW1, TPOAb^−^-LT_4_^−^-T3; AZ2, TPOAb^+^-LT_4_^+^-T2; AW2, TPOAb^+^-LT_4_^+^-T3; BZ2, TPOAb^−^-LT_4_^+^-T2; BW2, TPOAb^−^-LT_4_^+^-T3

From 264 fecal samples, 21,112,489 effective reads were obtained. A total of 17,777,893 high-quality reads, including 22,947 amplicon sequence variants (ASVs), were identified after sequence denoising or clustering (Fig. 1A). There were 329 common ASVs across the eight subgroups of TPOAb^+^/^−^ women with SCH stratified according to LT_4_ treatment status (Fig. 1A). The numbers of ASVs unique to T2 and T3 in each subgroup were as follows: TPOAb^+^ LT_4_^−^ (AZ1 or AW1) group, 1112 and 904, respectively; TPOAb^−^ LT_4_^−^ (BZ1 or BW1) group, 2392 and 1786, respectively; TPOAb^+^ LT_4_^+^ (AZ2 or AW2) group, 5204 and 3607, respectively; and TPOAb^−^ LT_4_^+^ (BZ2 or BW2) group, 4362 and 3251, respectively (Fig. 1A). A number of ASVs could be classified to the family (*n* = 3409), genus (*n* = 11,449) and species (*n* = 6815) levels (Fig. 1B). Taxonomic analysis showed that *Firmicutes* and *Bacteroidota* were the dominant phyla, followed by *Actinobacteriota* and *Proteobacteria* (Fig. 1C). *Bacteroides*, *Faecalibacterium*, *Bifidobacterium*, *Subdoligranulum*, *Prevotella*, *Lachnospira*, *Megamonas* and *Agathobacter* were the predominant genera (Fig. 1D). There were differences in ACE indices and Chao1 indices between T2 and T3 in the TPOAb^−^ LT_4_^−^ (*P* = 0.015 and 0.008, respectively), T2 and T3 TPOAb^+^ LT_4_^+^ (*P* = 0.004 and 0.008, respectively), T2 and T3 TPOAb^−^ LT_4_^+^ (*P* = 0.026 and 0.029, respectively) groups_._ There were no differences in ACE and Chao1 indices between T2 and T3 in the TPOAb^+^ LT_4_^−^ group. Furthermore, there were no differences in the Shannon and Simpson indices between T2 and T3 in the TPOAb^+^ LT_4_^−^, TPOAb^−^ LT_4_^−^, TPOAb^+^ LT_4_^+^, and TPOAb^−^ LT_4_^+^ groups (Fig. 2 and Supplementary Table 1).

**Fig. 3:**
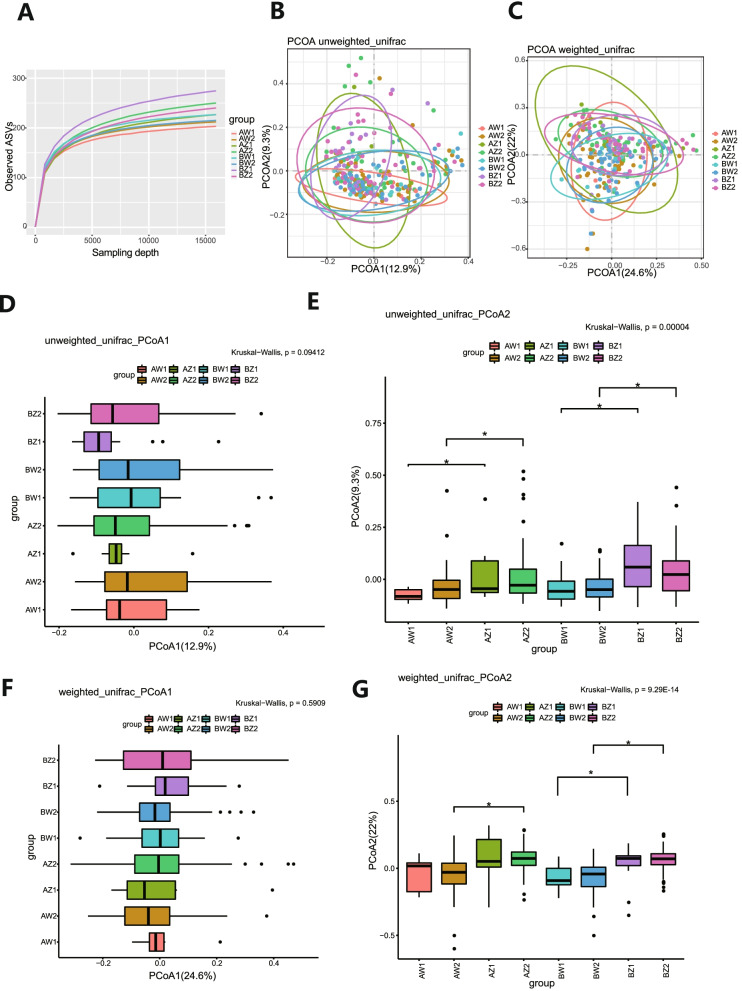
Rarefaction curves and β-diversity analysis. **A** Refraction curves based on random extraction of sequencing data from fecal samples from the eight subgroups. **B**, **D**-**E** β-diversity analysis conducted with the unweighted unifrac algorithm. **C**, **F**-**G** β-diversity analysis conducted with the weighted unifrac algorithm. * *P* < 0.05. AZ1, TPOAb^+^-LT_4_^−^-T2; AW1, TPOAb^+^-LT_4_^−^-T3; BZ1, TPOAb^−^-LT_4_^−^-T2; BW1, TPOAb^−^-LT_4_^−^-T3; AZ2, TPOAb^+^-LT_4_^+^-T2; AW2, TPOAb^+^-LT_4_^+^-T3; BZ2, TPOAb^−^-LT_4_^+^-T2; BW2, TPOAb^−^-LT_4_^+^-T3

The rarefaction curve of intestinal flora indicated that the number of ASVs analyzed was sufficient, and the distribution and abundance of species in each subgroup were high and adequate for data analysis (Fig. 3A). The results of PerMANOVA analysis were shown (Table 2). With respect to β-diversity, unweighted unifrac calculation revealed that PCoA1 and PCoA2 explained 12.9 and 9.3%, respectively, of the observed variation in the taxonomic profiles of intestinal microbiota between subgroups; β-diversity between T2 and T3 in the TPOAb^+^ LT_4_^−^ (PCoA2, *P* = 0.046)_,_ TPOAb^−^ LT_4_^−^ (PCoA2, *P* = 0.009)_,_ TPOAb^+^ LT_4_^+^ (PCoA2, *P* = 0.018), and TPOAb^−^ LT_4_^+^ (PCoA2, *P* = 0.001) groups were significantly different (Fig. 3B, D-E and Supplementary Table 2). However, weighted unifrac calculation revealed that PCoA1 and PCoA2 explained 24.6 and 22%, respectively, of the observed results; β-diversity between T2 and T3 in the TPOAb^−^ LT_4_^−^ (PCoA2, *P* = 0.001), TPOAb^+^ LT_4_^+^ (PCoA2, *P* = 7.79E-07), and TPOAb^−^ LT_4_^+^ (PCoA2, *P* = 1.92E-09) groups were significantly different; there were no significant differences in β-diversity between T2 and T3 in the TPOAb^+^ LT_4_^−^ group (Fig. 3C, F-G and Supplementary Table 3).

**Table 2:** The results of PerMANOVA

Distance	R^2^	*P* value
unweighted_unifrac	0.05972	**0.001**
weighted_unifrac	0.07626	**0.001**

R^2^ value represents the explanatory degree to the difference, *P* value indicates the reliability of this test. *P* < 0.05 was considered a statistically significant difference

### Dynamics of gut microbiota composition from T2 to T3

**Fig. 4:**
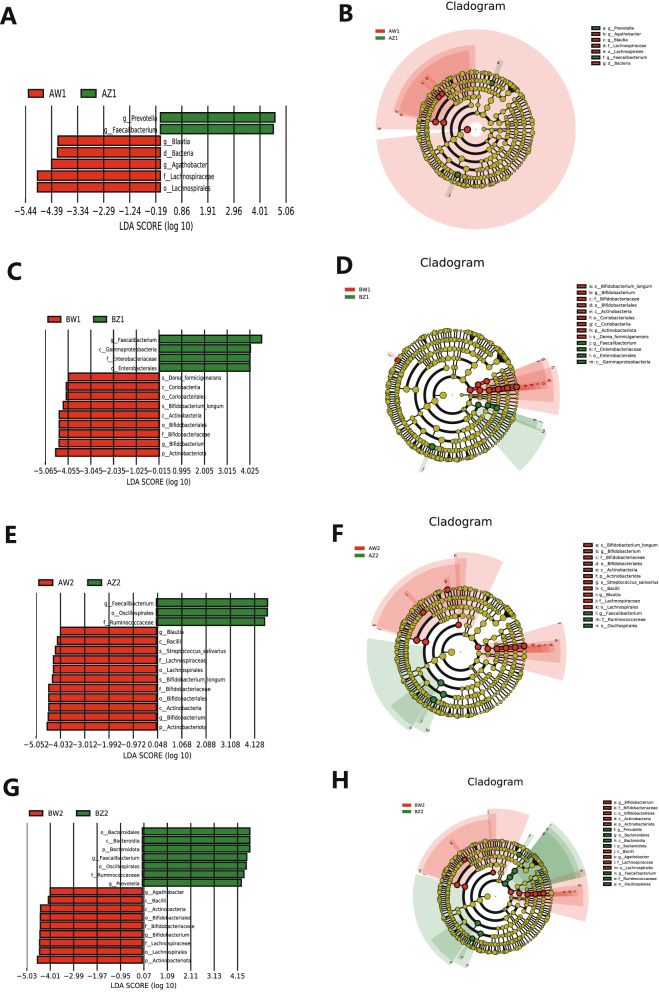
Intestinal microbiota as markers in TPOAb-positive women with SCH. **A**-**B** T2 vs. T3 in women with TPOAb-positive SCH and no LT_4_ treatment. **C**-**D** T2 vs. T3 in women with TPOAb-negative SCH and no LT_4_ treatment. **E**-**F** T2 vs. T3 in women with TPOAb-positive SCH and LT_4_ treatment. **G**-**H** T2 vs. T3 in women with TPOAb-negative SCH and LT_4_ treatment. LDA value distribution histogram: the green and red bars indicate higher abundance of intestinal microbiota in T2 and T3, respectively. Cladograms: circles radiating from the inside to the outside represent taxonomic levels from phylum to species. AZ1, TPOAb^+^-LT_4_^−^-T2; AW1, TPOAb^+^-LT_4_^−^-T3; BZ1, TPOAb^−^-LT_4_^−^-T2; BW1, TPOAb^−^-LT_4_^−^-T3; AZ2, TPOAb^+^-LT_4_^+^-T2; AW2, TPOAb^+^-LT_4_^+^-T3; BZ2, TPOAb^−^-LT_4_^+^-T2; BW2, TPOAb^−^-LT_4_^+^-T3

LEfSe analysis of differential species abundance was applied to identify intestinal microbiota that served as markers to distinguish TPOAb^+^ and TPOAb^−^ women with SCH during pregnancy. LEfSe analysis revealed 7 taxa showing different abundances between T2 and T3 in the TPOAb^+^ LT_4_^−^ group. Specifically, 2 and 5 taxa indicated greater and lesser abundances, respectively, in T2 than in T3. Moreover, 13 taxa were significantly different between T2 and T3 in the TPOAb^−^ LT_4_^−^ group. Specifically, 4 and 9 taxa showed greater and lesser abundances, respectively, in T2 than in T3. Intriguingly, genus *Faecalibacterium* had greater abundances in both these groups during T2 than during T3. The intestinal microbiota in the TPOAb^+^ LT_4_^−^ group were characterized by the enrichment of bacterial ASVs of the genus *Prevotella* in T2 and the kingdom *Bacteria*, order *Lachnospirales*, family *Lachnospiraceae*, and genera *Blautia* and *Agathobacter* in T3, as well as depletion of ASVs of the class *Gammaproteobacteria*, order *Enterobacterales*, and family *Enterobacteriaceae* in T2, and the phylum *Actinobacteriota*, classes *Coriobacteriia* and *Actinobacteria*, orders *Coriobacteriales* and *Bifidobacteriales*, family *Bifidobacteriaceae*, genus *Bifidobacterium*, and species *Dorea formicigenerans* and *Bifidobacterium longum* in T3 (Fig. 4A-D and Supplementary Table 4); the LEfSe analysis also revealed 14 taxa that showed different abundances between T2 and T3 in the TPOAb^+^ LT_4_^+^ group. Specifically, 3 and 11 taxa showed greater and lesser abundances, respectively, in T2 than in T3. Moreover, 16 taxa showed distinct abundances between T2 and T3 in the TPOAb^−^ LT_4_^+^ group. Specifically, 7 and 9 taxa exhibited greater and lesser abundances, respectively, in T2 than in T3. Intriguingly, 11 taxa showed consistent shifts in both groups. Specifically, 3 taxa (the order *Oscillospirales*, family *Ruminococcaceae*, and genus *Faecalibacterium*) showed greater abundances in both these groups in T2 than in T3, but 8 taxa (the phylum *Actinobacteriota*, classes *Bacilli* and *Actinobacteria*, orders *Bifidobacteriales* and *Lachnospirales*, families *Bifidobacteriaceae* and *Lachnospiraceae*, and genus *Bifidobacterium*) exhibited greater abundances in T3 than in T2. The intestinal microbiota in the TPOAb^+^ LT_4_^+^ group were characterized by the enrichment of bacterial ASVs of the genus *Blautia,* and species *Streptococcus salivarius* and *Bifidobacterium longum* in T3, and the depletion of ASVs of the phylum *Bacteroidota*, class *Bacteroidia*, order *Bacteroidales*, and genus *Prevotella* in T2 and the genus *Agathobacter* in T3 (Fig. 4E-H and Supplementary Table 5).

### Dynamics of gut microbiota function from T2 to T3

**Fig. 5:**
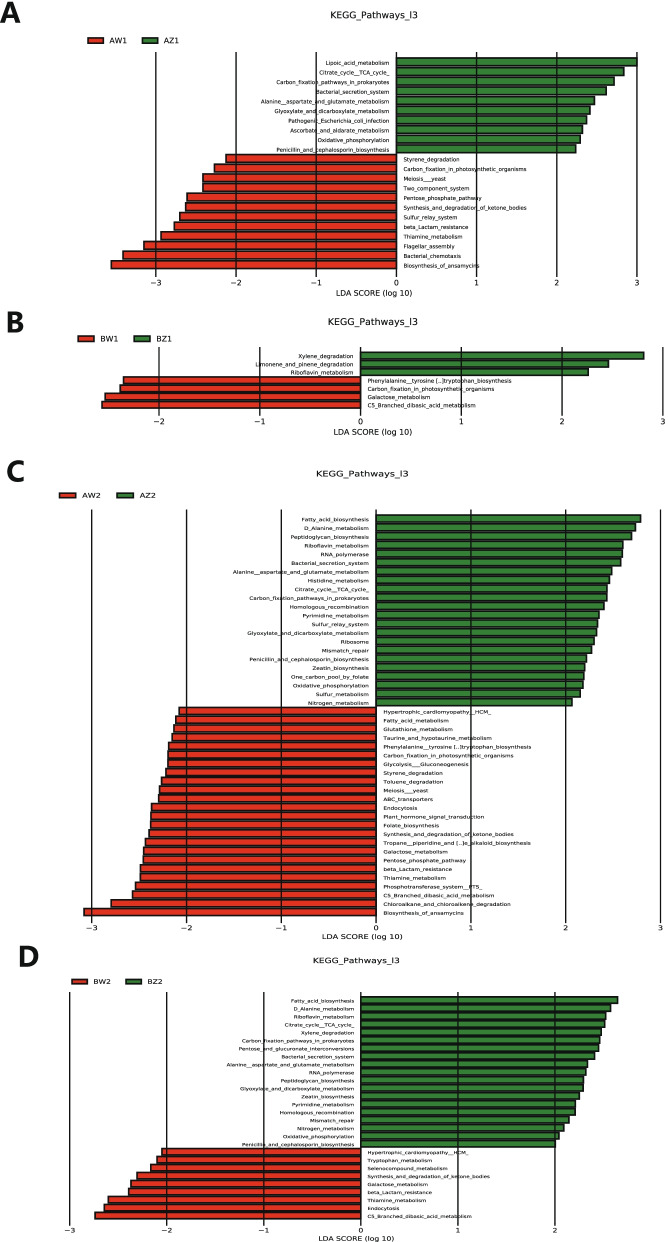
Functional profiling performed with PICRUSt2 based on the KEGG pathway map [43]. **A** T2 vs. T3 in women with TPOAb-positive SCH and no LT_4_ treatment. **B** T2 vs. T3 in women with TPOAb-negative SCH and no LT_4_ treatment. **C** T2 vs. T3 in women with TPOAb-positive SCH and LT_4_ treatment. **D** T2 vs. T3 in women with TPOAb-negative SCH and LT_4_ treatment. LDA value distribution histogram: the green and red bars indicate the metabolic functions with higher abundance in T2 and T3, respectively. AZ1, TPOAb^+^-LT_4_^−^-T2; AW1, TPOAb^+^-LT_4_^−^-T3; BZ1, TPOAb^−^-LT_4_^−^-T2; BW1, TPOAb^−^-LT_4_^−^-T3; AZ2, TPOAb^+^-LT_4_^+^-T2; AW2, TPOAb^+^-LT_4_^+^-T3; BZ2, TPOAb^−^-LT_4_^+^-T2; BW2, TPOAb^−^-LT_4_^+^-T3

LEfSe analysis of differential functional abundance based on the Kyoto Encyclopedia of Genes and Genomes (KEGG) pathway map can further predict the metabolic functions of the intestinal microbiota that serve as markers to distinguish TPOAb^+^ and TPOAb^−^ women with SCH during pregnancy. A total of 53 metabolic functions were discriminated between TPOAb^+^ and TPOAb^−^ women with SCH. The intestinal microbiota in the TPOAb^+^ LT_4_^−^ group were characterized by the enrichment of 10 metabolic functions (including alanine, aspartate and glutamate metabolism) in T2 and 11 metabolic functions (including pentose phosphate pathway) in T3 and by the depletion of 3 metabolic functions in T2, 3 metabolic functions (including galactose metabolism; phenylalanine, tyrosine, and tryptophan biosynthesis) in T3 (Fig. 5A, B and Supplementary Table 6). The intestinal microbiota in the TPOAb^+^ LT_4_^+^ group were characterized by the enrichment of 5 metabolic functions (including histidine metabolism) in T2 and 17 metabolic functions (including glycolysis and gluconeogenesis; pentose phosphate pathway; glutathione, taurine, and hypotaurine metabolism; phenylalanine, tyrosine, and tryptophan biosynthesis; fatty acid metabolism) in T3 and by the depletion of 2 metabolic functions (including pentose and glucuronate interconversions) in T2 and 2 metabolic functions (including tryptophan metabolism) in T3 (Fig. 5C, D and Supplementary Table 7).

## Discussion

In this single-center prospective cohort study, we described the dynamics of composition and metabolic function of intestinal microbiota in TPOAb^+^/^−^ women with SCH from T2 to T3. To our knowledge, this is the first study to have supported that women who were diagnosed with TPOAb^+^ SCH in T1 show distinct dynamics of gut microbiota from T2 to T3.

Previous studies have found that the frequency of female subjects with a family history of thyroid disease was higher in the SCH group than in the control group [26]. Our findings further indicated that TPOAb^+^ women with SCH are more likely to have a history of thyroid disease and show higher total cholesterol, high-density lipoprotein-cholesterol, and low-density lipoprotein-cholesterol levels in T1 than their TPOAb^−^ counterparts. However, the levels of these three parameters in TPOAb^+^/^−^ women with SCH in T1 were all within the normal range. It is well known that dietary habits have an effect on the composition of gut microbiota [27]. Therefore, this study minimized the influence of varying dietary habits across regions on intestinal microbiota. The baseline characteristics (such as ethnicity, culture and economic status) that affect dietary habits have shown no statistical differences. We concluded that women with a prior history of thyroid disease might be more likely to suffer from TPOAb^+^ SCH than those without such history.

The α-diversity reflects the microbial community diversity within the sample. While Chao1 index and ACE index measure species abundances, Shannon index and Simpson index are used to measure species diversities. The β-diversity analysis compares the degree of similarity in species diversity between different samples. In this study, unweighted and weighted unifrac calculations were used to analyze β-diversity. Unweighted unifrac calculation compares the presence or absence of species, while both species availability and species abundance need to be considered in weighted unifrac calculation. Intriguingly, inconsistent results of β-diversity, determined using unweighted and weighted unifrac calculations, between T2 and T3 in the TPOAb^+^ LT_4_^−^ group can be explained by the stricter definitions of weighted unifrac calculation. These findings provided the first evidence of diversity between T2 and T3 in women with TPOAb^+^/^−^ SCH.

The results from LEfSe analysis showed the apparently low taxonomic variability and high functional variability of gut microbiota from T2 to T3 in women with TPOAb^+^ SCH; this implies that dysbiosis of gut microbiota continued until late pregnancy. In the absence of LT_4_ treatment, we screened out six kinds of bacteria (genus *Prevotella* in T2 and kingdom *Bacteria*, order *Lachnospirales*, family *Lachnospiraceae*, and genera *Blautia* and *Agathobacter* in T3) as special bacteria for TPOAb^+^ women with SCH, and these were found to be involved in sugar and amino acid metabolism (alanine, aspartate, and glutamate metabolism; pentose phosphate pathway); in the presence of LT_4_ treatment, we distinguished three kinds of special bacteria for TPOAb^+^ women with SCH (genus *Blautia,* species *Streptococcus salivarius* and *Bifidobacterium longum* in T3), and these were found to be involved in sugar, lipid, and amino acid metabolism (glycolysis and gluconeogenesis; pentose phosphate pathway; fatty acid metabolism; phenylalanine, tyrosine, and tryptophan biosynthesis; glutathione, taurine, and hypotaurine metabolism; histidine metabolism). Especially, bacteria of genus *Blautia* were screened out as special bacteria for TPOAb^+^ LT_4_^+^/^−^ women. Bacteria of genus *Blautia* are obligate, anaerobic commensals that belong to family *Lachnospiraceae* [28]*.* Luu et al. discovered that the presence of bacteria of genus *Blautia* is negatively related to the prognosis of early-stage breast cancer [29]. Genus *Blautia*, which produces high levels of butyrate, was found to be more relevant to subjects with psoriasis than to non-psoriasis controls; however, this finding was not consistent with traditional observations in patients with inflammatory bowel diseases [30, 31]. Inflammation was positively linked with genus *Blautia* [32]. Remarkably, it has been reported that patients with cystic fibrosis were less likely to respond to inhaled aztreonam therapy when they showed abundance of genus *Prevotella* in the lung microbiome [33]. We predicted that genus *Prevotella* may affect the efficacy of LT_4_ drug therapy in patients with SCH. Genus *Prevotella* has been related to autoimmunity [34]. Moreover, genus *Agathobacter* were positively correlated with genus *Prevotella* [35]*.* In addition, abundance of family *Lachnospiraceae*, which belongs to order *Lachnospirales*, increased in subjects with gestational diabetes mellitus (GDM) in a previous study [36]; species *Streptococcus salivarius* was implicated in endophthalmitis [37]. However, species *Bifidobacterium longum* is probiotic, has an anti-inflammatory effect, and can be used to treat ulcerative colitis [38]. Therefore, findings of earlier studies support that these special bacteria, except for species *Bifidobacterium longum*, can be associated with diseases.

With respect to the absence of LT_4_ treatment, twelve kinds of bacteria (class *Gammaproteobacteria*, order Enterobacterales, and family *Enterobacteriaceae* in T2, phylum *Actinobacteriota*, classes *Coriobacteriia* and *Actinobacteria*, orders *Coriobacteriales* and *Bifidobacteriales*, family *Bifidobacteriaceae*, genus *Bifidobacterium*, and species *Dorea formicigenerans* and *Bifidobacterium longum* in T3) were considered as special bacteria for TPOAb^−^ women with SCH, and these were found to be involved in sugar and amino acid metabolism (galactose metabolism; phenylalanine, tyrosine, and tryptophan_biosynthesis). In the presence of LT_4_ treatment, five kinds of special bacteria were identified for TPOAb^−^ women with SCH (phylum *Bacteroidota*, class *Bacteroidia*, order *Bacteroidales*, and genus *Prevotella* in T2 and genus *Agathobacter* in T3), and these were found to be involved in sugar and amino acid metabolism (pentose and glucuronate interconversions; tryptophan metabolism). Earlier studies showed that subjects with GDM were characterized by a decrease in the abundance of members of family *Enterobacteriaceae* [36]*.* SCFAs, which are produced by species *Dorea formicigenerans*, may suppress the production of proinflammatory cytokines [39, 40]. Order *Coriobacteriales*, belonging to class *Coriobacteriia*, is characterized by lactic acid production [41]. Inflammation was negatively linked with genus *Bacteroides* [42]. Therefore, earlier study results support that these special bacteria, except for those belonging to genera *Prevotella* and *Agathobacter*, can be considered for the treatment of diseases.

In addition, we found that LT_4_ supplementation treatment can increase the abundance of beneficial bacteria (species *Bifidobacterium longum* in T3) in TPOAb^+^ women with SCH, which is consistent with the high recommendation of LT_4_ treatment for TPOAb^+^ women with SCH; however, LT_4_ supplementation can increase the abundance of harmful bacteria (genus *Prevotella* in T2 and genus *Agathobacter* in T3) in TPOAb^−^ women with SCH, which is consistent with the fact that LT_4_ treatment is appropriate recommended for TPOAb^−^ women with SCH. Although the roles and mechanisms of intestinal microbiota in pregnant TPOAb^+^ women with SCH remain to be elucidated, this study has suggested their involvement mainly in sugar, lipid, and amino acid metabolism. These findings provided the first evidence implying that composition and function of intestinal microbiota vary between women with TPOAb^+^ and TPOAb^−^ SCH from T2 to T3 in the presence or absence of LT_4_ treatment.

A limitation to this study is that it was conducted in a single center, and the normal ranges of TSH, FT_4_, and TPOAb in T1 varied from hospital to hospital. Another limitation is that dietary habits of pregnant women were not recorded in detail; their contribution to the gut microbiota of pregnant women could not be determined in the study. However, all the participants in this study have lived in Beijing. This study has minimized the influence of varying dietary habits across regions on gut microbiota.

## Conclusions

In conclusion, this single-center prospective cohort study found that women with TPOAb^+^ SCH exhibited a low gut microbiota variation and a high variation in its metabolic function from T2 to T3 in the presence or absence of LT_4_ treatment. The metabolic functions of different gut microbiota mainly included sugar, lipid, and amino acid metabolism. Changes in the abundances of three kinds of bacteria (species *Bifidobacterium longum* in T3, genus *Prevotella* in T2, and genus *Agathobacter* in T3) were abnormal in the presence of LT_4_ treatment. These findings suggest that gut microbiota can serve as potential therapeutic targets for TPOAb^+^ SCH during pregnancy. Further studies are needed to explore the causality between intestinal microbiota dynamics and TPOAb^+^ SCH and to, thereby, validate potential therapeutic targets.

## Supplementary Information

**Additional file 1: Supplementary Fig 1.** flow diagram TPO Ab-positive/negative women with SCH where stratified depending on whether or not they received LT_4_ treatment during pregnancy.

**Additional file 2: Supplementary Table 1.** α-diversity indexes. **Supplementary Table 2.** Principal coordinate analysis (PCoA1 and PCoA2) conducted with the unweighted unifrac algorithm. **Supplementary Table 3.** Principal coordinate analysis (PCoA1 and PCoA2) conducted with the weighted unifrac algorithm. **Supplementary Table 4.** LEfSe analysis of differential species abundance between AZ1 and AW1, between BZ1 and BW1. **Supplementary Table 5.** LEfSe analysis of differential species abundance between AZ2 and AW2, between BZ2 and BW2. **Supplementary Table 6.** LEfSe analysis of differential functional abundance between AZ1 and AW1, between BZ1 and BW1. **Supplementary Table 7.** LEfSe analysis of differential functional abundance between AZ2 and AW2, between BZ2 and BW2.

## Data Availability

The datasets generated and analysed during the current study are available in the NCBI repository, https://www.ncbi.nlm. nih.gov/, PRJNA751915. We want to keep the datasets private until acceptance. All data generated or analysed during this study are included in this article and its supplementary information files.

## Abbreviations

TPOAb: Anti-thyroid peroxidase antibody
T2: The second trimester
T3: The third trimester
SCH: Subclinical hypothyroidism
LT_4_: Levothyroxine
LDA: Linear discriminant analysis
LEfSe: Linear discriminant analysis effect size
ASVs: Amplicon sequence variants
ATA: American Thyroid Association
TSH: Thyroid stimulation hormone
FT_4_: Free T_4_
SCFAs: Short chain fatty acids
T1: The first trimester
PCoA1: The first principal coordinate axis
PCoA2: The second principal coordinate axis
PICRUSt: Phylogenetic Investigation of Communities by Reconstruction of Unobserved States
GDM: Gestational diabetes mellitus
