# Correction: Dynamics of gut microbiota during pregnancy in women with TPOAb-positive subclinical hypothyroidism: a prospective cohort study

**DOI:** 10.1186/s12884-022-04949-9

**Published:** 2022-08-06

**Authors:** Min Wu, Cheng Chi, Yuxi Yang, Shan Guo, Tianhe Li, Muqing Gu, Tingting Zhang, Huimin Gao, Ruixia Liu, Chenghong Yin

**Affiliations:** 1 Department of Internal Medicine, Beijing Obstetrics and Gynecology Hospital, Capital Medical University, Beijing Maternal and Child Health Care Hospital, Beijing, 100026 China; 2 School of Nursing, Jining Medical University, Jining, 272067 China; 3 Department of Central Laboratory, Beijing Obstetrics and Gynecology Hospital, Capital Medical University, Beijing Maternal and Child Health Care Hospital, Beijing, 100026 China

**Correction: BMC Pregnancy Childbirth 22, 592 (2022)**

**https://doi.org/10.1186/s12884-022-04923-5**

Following publication of the original article [1], the authors identified an error in the affiliation of Chenghong Yin.

The incorrect affiliation is: School of Nursing, Jining Medical University, Jining 272,067, China.

The correct affiliation is: Department of Central Laboratory, Beijing Obstetrics and Gynecology Hospital, Capital Medical University, Beijing Maternal and Child Health Care Hospital, Beijing 100,026, China.

The affiliation has been updated above and the original article [1] has been corrected.
