# Supplementary material for: Dynamics of gut microbiota during pregnancy in women with TPOAb-positive subclinical hypothyroidism: a prospective cohort study
Source: BMC Pregnancy Childbirth. 2022 Jul 26;22:592. doi: 10.1186/s12884-022-04923-5 (PMC9316685; doi:10.1186/s12884-022-04923-5)
Supplement: Supplementary file 1 — Additional file 1: Supplementary Fig 1. flow diagram TPO Ab-positive/negative women with SCH where stratified depending on whether or not they received LT4 treatment during pregnancy. [file 12884_2022_4923_MOESM1_ESM.pdf]

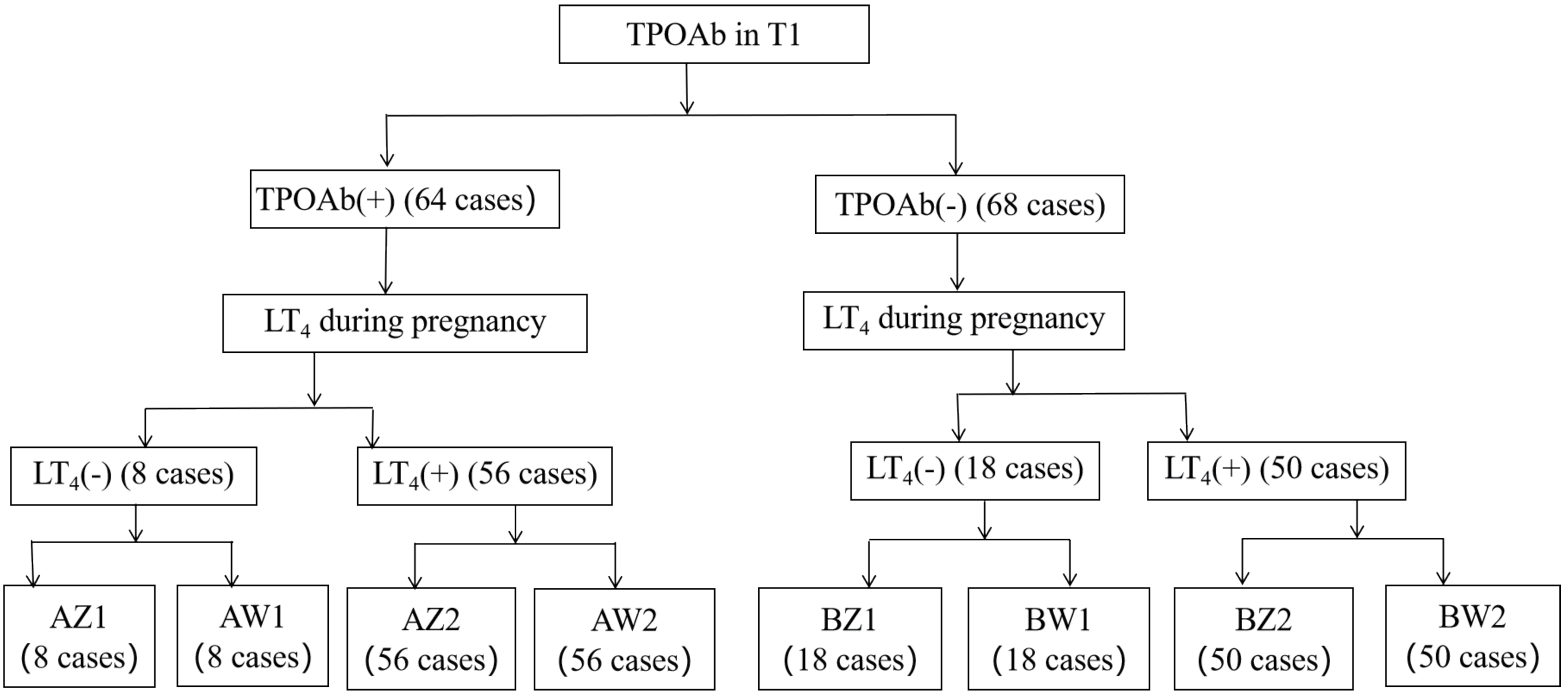

Flow diagram

AZ1, TPOAb(+)-LT_4(−)-T2; AW1, TPOAb(+)-LT_4(−)-T3;

AZ2, TPOAb(+)-LT_4(+)-T2; AW2, TPOAb(+)-LT_4(+)-T3;

BZ1, TPOAb(−)-LT_4(−)-T2; BW1, TPOAb(−)-LT_4(−)-T3;

BZ2, TPOAb(−)-LT_4(+)-T2; BW2, TPOAb(−)-LT_4(+)-T3.

Supplementary Fig. 1 Flow diagram. TPOAb-positive/negative women with SCH were stratified depending on whether or not they received LT_4 treatment during pregnancy.
