# Supplementary material for: Dynamics of gut microbiota during pregnancy in women with TPOAb-positive subclinical hypothyroidism: a prospective cohort study
Source: BMC Pregnancy Childbirth. 2022 Jul 26;22:592. doi: 10.1186/s12884-022-04923-5 (PMC9316685; doi:10.1186/s12884-022-04923-5)
Supplement: Supplementary file 2 — Additional file 2: Supplementary Table 1. α-diversity indexes. Supplementary Table 2. Principal coordinate analysis (PCoA1 and PCoA2) conducted with the unweighted unifrac algorithm. Supplementary Table 3. Principal coordinate analysis (PCoA1 and PCoA2) conducted with the weighted unifrac algorithm. Supplementary Table 4. LEfSe analysis of differential species abundance between AZ1 and AW1, between BZ1 and BW1. Supplementary Table 5. LEfSe analysis of differential species abundance between AZ2 and AW2, between BZ2 and BW2. Supplementary Table 6. LEfSe analysis of differential functional abundance between AZ1 and AW1, between BZ1 and BW1. Supplementary Table 7. LEfSe analysis of differential functional abundance between AZ2 and AW2, between BZ2 and BW2. [file 12884_2022_4923_MOESM2_ESM.docx]

**Supplementary Table 1** α-diversity indexes

| α-diversity index | AZ1_AW1_p.value | BZ1_BW1_p.value | AZ2_AW2_p.value | BZ2_BW2_p.value |
| --- | --- | --- | --- | --- |
| ACE | 0.400814 | **0.014844** | **0.004196** | **0.025967** |
| Chao1 | 0.462250 | **0.007869** | **0.007896** | **0.029373** |
| Shannon | 0.344562 | 0.974760 | 0.176998 | 0.149639 |
| Simpson | 0.293622 | 0.949546 | 0.242138 | 0.068764 |

AZ1, TPOAb^+^-LT_4_^−^-T2, women in T2 with TPOAb-positive SCH and no LT_4_ treatment; AW1, TPOAb^+^-LT_4_^−^-T3, women in T3 with TPOAb-positive SCH and no LT_4_ treatment; BZ1, TPOAb^−^-LT_4_^−^-T2, women in T2 with TPOAb-negative SCH and no LT_4_ treatment; BW1, TPOAb^−^-LT_4_^−^-T3, women in T3 with TPOAb-negative SCH and no LT_4_ treatment; AZ2, TPOAb^+^-LT_4_^+^-T2, women in T2 with TPOAb-positive SCH and LT_4_ treatment; AW2, TPOAb^+^-LT_4_^+^-T3, women in T3 with TPOAb-positive SCH and LT_4_ treatment; BZ2, TPOAb^−^-LT_4_^+^-T2, women in T2 with TPOAb-negative SCH and LT_4_ treatment; BW2, TPOAb^−^-LT_4_^+^-T3, women in T3 with TPOAb-negative SCH and LT_4_ treatment. *P* < 0.05 was considered a statistically significant difference.

**Supplementary Table 2** Principal coordinate analysis (PCoA1 and PCoA2) conducted with the unweighted unifrac algorithm

| Group1 | Group2 | PCoA1_p.value | PCoA2_p.value |
| --- | --- | --- | --- |
| AZ1 | AW1 | 0.833635 | **0.045999** |
| BZ1 | BW1 | 0.093573 | **0.008639** |
| AZ2 | AW2 | 0.098402 | **0.017865** |
| BZ2 | BW2 | 0.138295 | **0.000695** |

AZ1, TPOAb^+^-LT4^−^-T2; AW1, TPOAb^+^-LT4^−^-T3; BZ1, TPOAb^−^-LT4^−^-T2; BW1, TPOAb^−^-LT4^−^-T3; AZ2, TPOAb^+^-LT4^+^-T2; AW2, TPOAb^+^-LT4^+^-T3; BZ2, TPOAb^−^-LT4^+^-T2; BW2, TPOAb^−^-LT4^+^-T3. *P* < 0.05 was considered a statistically significant difference.

**Supplementary Table 3** Principal coordinate analysis (PCoA1 and PCoA2) conducted with the weighted unifrac algorithm

| Group1 | Group2 | PCoA1_p.value | PCoA2_p.value |
| --- | --- | --- | --- |
| AZ1 | AW1 | 0.528612 | 0.172167 |
| BZ1 | BW1 | 0.282056 | **0.000894** |
| AZ2 | AW2 | 0.160788 | **7.79E-07** |
| BZ2 | BW2 | 0.751156 | **1.92E-09** |

AZ1, TPOAb^+^-LT4^−^-T2; AW1, TPOAb^+^-LT4^−^-T3; BZ1, TPOAb^−^-LT4^−^-T2; BW1, TPOAb^−^-LT4^−^-T3; AZ2, TPOAb^+^-LT4^+^-T2; AW2, TPOAb^+^-LT4^+^-T3; BZ2, TPOAb^−^-LT4^+^-T2; BW2, TPOAb^−^-LT4^+^-T3. *P* < 0.05 was considered a statistically significant difference.

**Supplementary Table 4** LEfSe analysis of differential species abundance between AZ1 and AW1, between BZ1 and BW1

| Feature | log(max) | Group | LDA | p.value |
| --- | --- | --- | --- | --- |
| d__Bacteria.p__Bacteroidota.c__Bacteroidia.o__Bacteroidales.f__Prevotellaceae.g__Prevotella | 4.857211 | AZ1 | 4.616520 | **0.015873** |
| d__Bacteria.p__Firmicutes.c__Clostridia.o__Lachnospirales | 5.569102 | AW1 | 4.956853 | **0.004574** |
| d__Bacteria | 6.000000 | AW1 | 4.149800 | **0.027083** |
| d__Bacteria.p__Firmicutes.c__Clostridia.o__Lachnospirales.f__Lachnospiraceae.g__Agathobacter | 4.787014 | AW1 | 4.386174 | **0.031200** |
| d__Bacteria.p__Firmicutes.c__Clostridia.o__Oscillospirales.f__Ruminococcaceae.g__Faecalibacterium | 5.215564 | AZ1 | 4.551723 | **0.045999** |
| d__Bacteria.p__Firmicutes.c__Clostridia.o__Lachnospirales.f__Lachnospiraceae.g__Blautia | 4.549264 | AW1 | 4.125094 | **0.002322** |
| d__Bacteria.p__Firmicutes.c__Clostridia.o__Lachnospirales.f__Lachnospiraceae | 5.569116 | AW1 | 4.956464 | **0.004574** |
| d__Bacteria.p__Proteobacteria.c__Gammaproteobacteria.o__Enterobacterales | 4.408002 | BZ1 | 4.028922 | **0.001929** |
| d__Bacteria.p__Actinobacteriota.c__Actinobacteria | 5.050549 | BW1 | 4.446406 | **0.003988** |
| d__Bacteria.p__Actinobacteriota.c__Actinobacteria.o__Bifidobacteriales | 5.046329 | BW1 | 4.447017 | **0.003257** |
| d__Bacteria.p__Proteobacteria.c__Gammaproteobacteria.o__Enterobacterales.f__Enterobacteriaceae | 4.406648 | BZ1 | 4.031879 | **0.000893** |
| d__Bacteria.p__Actinobacteriota.c__Coriobacteriia.o__Coriobacteriales | 4.524558 | BW1 | 4.137096 | **0.001396** |
| d__Bacteria.p__Actinobacteriota.c__Actinobacteria.o__Bifidobacteriales.f__Bifidobacteriaceae.g__Bifidobacterium | 5.062014 | BW1 | 4.459319 | **0.002939** |
| d__Bacteria.p__Actinobacteriota.c__Coriobacteriia | 4.524430 | BW1 | 4.136967 | **0.001396** |
| d__Bacteria.p__Actinobacteriota.c__Actinobacteria.o__Bifidobacteriales.f__Bifidobacteriaceae.g__Bifidobacterium.s__Bifidobacterium_longum | 4.895784 | BW1 | 4.272288 | **0.016783** |
| d__Bacteria.p__Actinobacteriota | 5.163830 | BW1 | 4.607456 | **0.002387** |
| d__Bacteria.p__Firmicutes.c__Clostridia.o__Oscillospirales.f__Ruminococcaceae.g__Faecalibacterium | 5.182139 | BZ1 | 4.545621 | **0.020909** |
| d__Bacteria.p__Proteobacteria.c__Gammaproteobacteria | 4.541756 | BZ1 | 4.049519 | **0.004407** |
| d__Bacteria.p__Actinobacteriota.c__Actinobacteria.o__Bifidobacteriales.f__Bifidobacteriaceae | 5.046409 | BW1 | 4.447038 | **0.003257** |
| d__Bacteria.p__Firmicutes.c__Clostridia.o__Lachnospirales.f__Lachnospiraceae.g__Dorea.s__Dorea_formicigenerans | 4.409730 | BW1 | 4.038655 | **0.005881** |

AZ1, TPOAb^+^-LT4^−^-T2; AW1, TPOAb^+^-LT4^−^-T3; BZ1, TPOAb^−^-LT4^−^-T2; BW1, TPOAb^−^-LT4^−^-T3. *P* < 0.05 was considered a statistically significant difference.

**Supplementary Table 5** LEfSe analysis of differential species abundance between AZ2 and AW2, between BZ2 and BW2

| Feature | log(max) | Group | LDA | p.value |
| --- | --- | --- | --- | --- |
| d__Bacteria.p__Actinobacteriota.c__Actinobacteria | 5.012463 | AW2 | 4.521629 | **0.001344** |
| d__Bacteria.p__Firmicutes.c__Bacilli.o__Lactobacillales.f__Streptococcaceae.g__Streptococcus.s__Streptococcus_salivarius | 4.893299 | AW2 | 4.241382 | **0.002498** |
| d__Bacteria.p__Actinobacteriota.c__Actinobacteria.o__Bifidobacteriales | 5.003616 | AW2 | 4.521580 | **0.001239** |
| d__Bacteria.p__Actinobacteriota.c__Actinobacteria.o__Bifidobacteriales.f__Bifidobacteriaceae.g__Bifidobacterium | 5.023204 | AW2 | 4.542555 | **0.001142** |
| d__Bacteria.p__Firmicutes.c__Bacilli | 4.763587 | AW2 | 4.158105 | **0.000001** |
| d__Bacteria.p__Firmicutes.c__Clostridia.o__Lachnospirales | 5.454825 | AW2 | 4.324948 | **0.022888** |
| d__Bacteria.p__Actinobacteriota | 5.126994 | AW2 | 4.589667 | **0.000066** |
| d__Bacteria.p__Firmicutes.c__Clostridia.o__Oscillospirales.f__Ruminococcaceae.g__Faecalibacterium | 5.243442 | AZ2 | 4.666269 | **0.000000** |
| d__Bacteria.p__Firmicutes.c__Clostridia.o__Oscillospirales.f__Ruminococcaceae | 5.360481 | AZ2 | 4.543204 | **0.000055** |
| d__Bacteria.p__Firmicutes.c__Clostridia.o__Lachnospirales.f__Lachnospiraceae.g__Blautia | 4.442440 | AW2 | 4.028313 | **0.000000** |
| d__Bacteria.p__Firmicutes.c__Clostridia.o__Oscillospirales | 5.452957 | AZ2 | 4.659470 | **0.000030** |
| d__Bacteria.p__Firmicutes.c__Clostridia.o__Lachnospirales.f__Lachnospiraceae | 5.454636 | AW2 | 4.323861 | **0.025066** |
| d__Bacteria.p__Actinobacteriota.c__Actinobacteria.o__Bifidobacteriales.f__Bifidobacteriaceae.g__Bifidobacterium.s__Bifidobacterium_longum | 4.811726 | AW2 | 4.368928 | **0.000589** |
| d__Bacteria.p__Actinobacteriota.c__Actinobacteria.o__Bifidobacteriales.f__Bifidobacteriaceae | 5.003714 | AW2 | 4.521508 | **0.001239** |
| d__Bacteria.p__Bacteroidota.c__Bacteroidia.o__Bacteroidales.f__Prevotellaceae.g__Prevotella | 4.898758 | BZ2 | 4.310506 | **0.045923** |
| d__Bacteria.p__Actinobacteriota.c__Actinobacteria | 5.028494 | BW2 | 4.429882 | **0.001195** |
| d__Bacteria.p__Actinobacteriota.c__Actinobacteria.o__Bifidobacteriales | 5.023642 | BW2 | 4.437346 | **0.001348** |
| d__Bacteria.p__Firmicutes.c__Clostridia.o__Lachnospirales.f__Lachnospiraceae.g__Agathobacter | 4.572714 | BW2 | 4.014211 | **0.013397** |
| d__Bacteria.p__Bacteroidota.c__Bacteroidia | 5.411632 | BZ2 | 4.682049 | **0.003317** |
| d__Bacteria.p__Firmicutes.c__Bacilli | 4.715182 | BW2 | 4.090399 | **0.000170** |
| d__Bacteria.p__Firmicutes.c__Clostridia.o__Lachnospirales | 5.474032 | BW2 | 4.473233 | **0.001448** |
| d__Bacteria.p__Bacteroidota | 5.411606 | BZ2 | 4.681987 | **0.003317** |
| d__Bacteria.p__Actinobacteriota.c__Actinobacteria.o__Bifidobacteriales.f__Bifidobacteriaceae.g__Bifidobacterium | 5.041497 | BW2 | 4.456356 | **0.001239** |
| d__Bacteria.p__Bacteroidota.c__Bacteroidia.o__Bacteroidales | 5.411604 | BZ2 | 4.682396 | **0.003316** |
| d__Bacteria.p__Actinobacteriota | 5.144633 | BW2 | 4.566781 | **0.000001** |
| d__Bacteria.p__Firmicutes.c__Clostridia.o__Oscillospirales.f__Ruminococcaceae.g__Faecalibacterium | 5.199536 | BZ2 | 4.549184 | **0.000416** |
| d__Bacteria.p__Firmicutes.c__Clostridia.o__Oscillospirales.f__Ruminococcaceae | 5.327129 | BZ2 | 4.427405 | **0.025059** |
| d__Bacteria.p__Firmicutes.c__Clostridia.o__Oscillospirales | 5.423290 | BZ2 | 4.525446 | **0.017390** |
| d__Bacteria.p__Firmicutes.c__Clostridia.o__Lachnospirales.f__Lachnospiraceae | 5.473989 | BW2 | 4.472935 | **0.001448** |
| d__Bacteria.p__Actinobacteriota.c__Actinobacteria.o__Bifidobacteriales.f__Bifidobacteriaceae | 5.023699 | BW2 | 4.437349 | **0.001348** |

AZ2, TPOAb^+^-LT4^+^-T2; AW2, TPOAb^+^-LT4^+^-T3; BZ2, TPOAb^−^-LT4^+^-T2; BW2, TPOAb^−^-LT4^+^-T3. *P* < 0.05 was considered a statistically significant difference.

**Supplementary Table 6** LEfSe analysis of differential functional abundance between AZ1 and AW1, between BZ1 and BW1

| Feature | log(max) | Group | LDA | p.value |
| --- | --- | --- | --- | --- |
| Glyoxylate_and_dicarboxylate_metabolism | 3.885987 | AZ1 | 2.413061 | **0.027423** |
| Lipoic_acid_metabolism | 3.740586 | AZ1 | 2.999956 | **0.035692** |
| Carbon_fixation_pathways_in_prokaryotes | 4.033169 | AZ1 | 2.714787 | **0.015714** |
| Synthesis_and_degradation_of_ketone_bodies | 3.475141 | AW1 | 2.627618 | **0.045999** |
| Biosynthesis_of_ansamycins | 4.746749 | AW1 | 3.555958 | **0.006323** |
| Two_component_system | 3.597616 | AW1 | 2.412607 | **0.004574** |
| Meiosis___yeast | 0.561014 | AW1 | 2.412186 | **0.004229** |
| Citrate_cycle__TCA_cycle_ | 3.962682 | AZ1 | 2.835219 | **0.015714** |
| Bacterial_secretion_system | 3.947451 | AZ1 | 2.616327 | **0.015714** |
| Penicillin_and_cephalosporin_biosynthesis | 3.008494 | AZ1 | 2.236152 | **0.045999** |
| Carbon_fixation_in_photosynthetic_organisms | 4.205726 | AW1 | 2.269469 | **0.045999** |
| Pathogenic_Escherichia_coli_infection | 1.490267 | AZ1 | 2.371614 | **0.011109** |
| Alanine__aspartate_and_glutamate_metabolism | 4.282104 | AZ1 | 2.469057 | **0.015714** |
| Flagellar_assembly | 3.942473 | AW1 | 3.148621 | **0.035692** |
| Oxidative_phosphorylation | 3.686486 | AZ1 | 2.291935 | **0.015714** |
| Sulfur_relay_system | 4.083486 | AW1 | 2.701047 | **0.027423** |
| Ascorbate_and_aldarate_metabolism | 3.352124 | AZ1 | 2.319259 | **0.027423** |
| Styrene_degradation | 2.580823 | AW1 | 2.124683 | **0.019931** |
| Thiamine_metabolism | 4.245962 | AW1 | 2.934755 | **0.027423** |
| Bacterial_chemotaxis | 4.170541 | AW1 | 3.408390 | **0.008652** |
| Pentose_phosphate_pathway | 4.287327 | AW1 | 2.610128 | **0.045999** |
| beta_Lactam_resistance | 3.453594 | AW1 | 2.770251 | **0.002322** |
| Galactose_metabolism | 4.105920 | BW1 | 2.533516 | **0.010385** |
| Phenylalanine__tyrosine_and_tryptophan_biosynthesis | 4.122842 | BW1 | 2.351875 | **0.046236** |
| C5_Branched_dibasic_acid_metabolism | 4.276751 | BW1 | 2.564486 | **0.016193** |
| Limonene_and_pinene_degradation | 2.818382 | BZ1 | 2.459200 | **0.004173** |
| Riboflavin_metabolism | 3.973303 | BZ1 | 2.258454 | **0.029031** |
| Carbon_fixation_in_photosynthetic_organisms | 4.204346 | BW1 | 2.384318 | **0.049810** |
| Xylene_degradation | 3.181574 | BZ1 | 2.809332 | **0.001139** |

AZ1, TPOAb^+^-LT4^−^-T2; AW1, TPOAb^+^-LT4^−^-T3; BZ1, TPOAb^−^-LT4^−^-T2; BW1, TPOAb^−^-LT4^−^-T3. *P* < 0.05 was considered a statistically significant difference.

**Supplementary Table 7** LEfSe analysis of differential functional abundance between AZ2 and AW2, between BZ2 and BW2

| Feature | log(max) | Group | LDA | p.value |
| --- | --- | --- | --- | --- |
| Ribosome | 4.220748 | AZ2 | 2.299565 | **0.037226** |
| Glyoxylate_and_dicarboxylate_metabolism | 3.879066 | AZ2 | 2.325792 | **0.000015** |
| Fatty_acid_metabolism | 3.540557 | AW2 | 2.112889 | **0.023955** |
| Taurine_and_hypotaurine_metabolism | 3.761858 | AW2 | 2.151254 | **0.002834** |
| Hypertrophic_cardiomyopathy__HCM_ | 0.644633 | AW2 | 2.077852 | **0.000000** |
| Endocytosis | 0.314551 | AW2 | 2.368111 | **0.008649** |
| Homologous_recombination | 4.204596 | AZ2 | 2.402728 | **0.000361** |
| Mismatch_repair | 4.244082 | AZ2 | 2.272044 | **0.002478** |
| Toluene_degradation | 2.931433 | AW2 | 2.262754 | **0.017485** |
| Folate_biosynthesis | 3.989196 | AW2 | 2.378490 | **0.038846** |
| Carbon_fixation_pathways_in_prokaryotes | 4.020400 | AZ2 | 2.434125 | **0.000246** |
| Peptidoglycan_biosynthesis | 4.321005 | AZ2 | 2.694235 | **0.000252** |
| Synthesis_and_degradation_of_ketone_bodies | 3.499320 | AW2 | 2.394582 | **0.019319** |
| Biosynthesis_of_ansamycins | 4.715412 | AW2 | 3.080363 | **0.012963** |
| Galactose_metabolism | 4.109653 | AW2 | 2.451874 | **0.012963** |
| Meiosis___yeast | 0.975924 | AW2 | 2.284254 | **0.000000** |
| Tropane__piperidine_and_pyridine_alkaloid_biosynthesis | 3.550039 | AW2 | 2.433733 | **0.021323** |
| Nitrogen_metabolism | 3.805282 | AZ2 | 2.066120 | **0.036179** |
| Pyrimidine_metabolism | 4.092320 | AZ2 | 2.350934 | **0.000044** |
| Citrate_cycle__TCA_cycle_ | 3.945302 | AZ2 | 2.435378 | **0.003115** |
| Phenylalanine__tyrosine_and_tryptophan_biosynthesis | 4.117530 | AW2 | 2.188434 | **0.007432** |
| One_carbon_pool_by_folate | 4.257864 | AZ2 | 2.190832 | **0.037760** |
| Bacterial_secretion_system | 3.939709 | AZ2 | 2.580213 | **0.000000** |
| Fatty_acid_biosynthesis | 4.267603 | AZ2 | 2.789923 | **0.000163** |
| Zeatin_biosynthesis | 3.813889 | AZ2 | 2.200144 | **0.002431** |
| Glutathione_metabolism | 3.631081 | AW2 | 2.133047 | **0.047217** |
| D_Alanine_metabolism | 4.261543 | AZ2 | 2.735109 | **0.000002** |
| Histidine_metabolism | 4.158848 | AZ2 | 2.460546 | **0.000141** |
| C5_Branched_dibasic_acid_metabolism | 4.276533 | AW2 | 2.568948 | **0.001096** |
| Glycolysis___Gluconeogenesis | 4.064595 | AW2 | 2.196386 | **0.004944** |
| Penicillin_and_cephalosporin_biosynthesis | 2.956739 | AZ2 | 2.218554 | **0.000002** |
| Plant_hormone_signal_transduction | 0.222003 | AW2 | 2.375159 | **0.024393** |
| ABC_transporters | 3.880417 | AW2 | 2.293783 | **0.019020** |
| Alanine__aspartate_and_glutamate_metabolism | 4.276266 | AZ2 | 2.485334 | **0.000547** |
| Sulfur_metabolism | 3.926828 | AZ2 | 2.153231 | **0.003552** |
| Riboflavin_metabolism | 3.983608 | AZ2 | 2.602551 | **0.000000** |
| Carbon_fixation_in_photosynthetic_organisms | 4.205929 | AW2 | 2.194298 | **0.004274** |
| Oxidative_phosphorylation | 3.670109 | AZ2 | 2.182221 | **0.000025** |
| Chloroalkane_and_chloroalkene_degradation | 3.555275 | AW2 | 2.794683 | **0.006296** |
| Phosphotransferase_system__PTS_ | 3.675973 | AW2 | 2.537956 | **0.001291** |
| Sulfur_relay_system | 4.057522 | AZ2 | 2.335417 | **0.018434** |
| Styrene_degradation | 2.789956 | AW2 | 2.217411 | **0.000005** |
| Thiamine_metabolism | 4.233080 | AW2 | 2.486855 | **0.011941** |
| Pentose_phosphate_pathway | 4.276997 | AW2 | 2.455977 | **0.001517** |
| RNA_polymerase | 4.101725 | AZ2 | 2.597033 | **0.001317** |
| beta_Lactam_resistance | 3.407735 | AW2 | 2.486178 | **0.000502** |
| Glyoxylate_and_dicarboxylate_metabolism | 3.880704 | BZ2 | 2.289950 | **0.000220** |
| Hypertrophic_cardiomyopathy__HCM_ | 0.717633 | BW2 | 2.049574 | **0.000002** |
| Endocytosis | 0.000000 | BW2 | 2.642741 | **0.017147** |
| Homologous_recombination | 4.202059 | BZ2 | 2.207503 | **0.006074** |
| Mismatch_repair | 4.242211 | BZ2 | 2.143269 | **0.019083** |
| Carbon_fixation_pathways_in_prokaryotes | 4.020658 | BZ2 | 2.461142 | **0.000913** |
| Peptidoglycan_biosynthesis | 4.314087 | BZ2 | 2.292700 | **0.036723** |
| Synthesis_and_degradation_of_ketone_bodies | 3.487968 | BW2 | 2.306066 | **0.039941** |
| Galactose_metabolism | 4.109969 | BW2 | 2.368240 | **0.015240** |
| Nitrogen_metabolism | 3.799884 | BZ2 | 2.090431 | **0.000827** |
| Pyrimidine_metabolism | 4.089530 | BZ2 | 2.209958 | **0.005128** |
| Citrate_cycle__TCA_cycle_ | 3.946845 | BZ2 | 2.512479 | **0.003871** |
| Bacterial_secretion_system | 3.932151 | BZ2 | 2.408181 | **0.000512** |
| Fatty_acid_biosynthesis | 4.261055 | BZ2 | 2.644565 | **0.004411** |
| Zeatin_biosynthesis | 3.814563 | BZ2 | 2.250261 | **0.033729** |
| D_Alanine_metabolism | 4.260029 | BZ2 | 2.572356 | **0.001224** |
| C5_Branched_dibasic_acid_metabolism | 4.277270 | BW2 | 2.737735 | **0.000203** |
| Penicillin_and_cephalosporin_biosynthesis | 2.919319 | BZ2 | 2.003355 | **0.002258** |
| Pentose_and_glucuronate_interconversions | 3.939405 | BZ2 | 2.448692 | **0.004133** |
| Alanine__aspartate_and_glutamate_metabolism | 4.272659 | BZ2 | 2.334113 | **0.004133** |
| Riboflavin_metabolism | 3.985363 | BZ2 | 2.522641 | **0.000288** |
| Oxidative_phosphorylation | 3.665471 | BZ2 | 2.040068 | **0.007477** |
| Xylene_degradation | 3.009491 | BZ2 | 2.475896 | **0.002487** |
| Selenocompound_metabolism | 4.022907 | BW2 | 2.163892 | **0.011405** |
| Tryptophan_metabolism | 3.172082 | BW2 | 2.099236 | **0.035644** |
| Thiamine_metabolism | 4.236573 | BW2 | 2.601745 | **0.002258** |
| RNA_polymerase | 4.094127 | BZ2 | 2.317549 | **0.030414** |
| beta_Lactam_resistance | 3.397741 | BW2 | 2.391956 | **0.001483** |

AZ2, TPOAb^+^-LT4^+^-T2; AW2, TPOAb^+^-LT4^+^-T3; BZ2, TPOAb^−^-LT4^+^-T2; BW2, TPOAb^−^-LT4^+^-T3. *P* < 0.05 was considered a statistically significant difference.
